# FAMily Motivational Engagement Strategy (FAMES) for coordinated specialty care programs: study protocol to evaluate a culturally responsive engagement intervention and equity focused implementation strategies in a hybrid type 2 randomized stepped-wedge trial

**DOI:** 10.1186/s13063-025-09280-0

**Published:** 2025-12-16

**Authors:** Oladunni Oluwoye, Bryony Stokes, Annette S. Crisanti, Karina Silva Garcia, Liat Kriegel, Megan Puzia, Amanda L. Sanchez, Rachel C. Shelton, Douglas L. Weeks

**Affiliations:** 1 Department of Community and Behavioral Health, Elson S. Floyd College of Medicine, Washington State University, Spokane, WA 99210-1495 USA; 2 Department of Psychiatry, University of New Mexico, Albuquerque, NM USA; 3 Department of Psychology, George Mason University, Fairfax, USA; 4 Mailman School of Public Health, Columbia University, New York, NY USA

**Keywords:** Coordinated specialty care, Equity, Engagement, Family peers, Family members, Mental health services, Psychosis

## Abstract

**Background:**

Coordinated specialty care (CSC) models for first episode psychosis include evidence-informed family education and support as a core component of care. Evidence suggests low rates of family engagement in many CSC programs. To address this gap, the FAMily Motivational Engagement Strategy (FAMES), a culturally responsive intervention, was previously developed, pilot-tested, and found to positively impact family engagement in CSC. The goals of this Hybrid Type 2 Effectiveness-Implementation study are to investigate whether FAMES improves family engagement in CSC, assess activation of target mechanisms, and evaluate a Culturally Responsive Approach to Targeting Equity (CURATE) implementation package used to support the integration of FAMES in CSC.

**Methods:**

A stepped-wedge trial will be conducted with nine CSC sites, each randomized to one of three waves. CSC sites will initially recruit participant dyads into an attention control condition (*n* = 225) followed by a separate cohort of dyads who will receive FAMES (*n* = 225). Primary (engagement) and secondary (perceived stress, anxiety) outcomes, as well as mechanisms (self-efficacy, connectedness, motivation) among family members will be assessed. Secondary outcomes will also include client-level outcomes such as symptoms and engagement. Guided by the Reach, Effectiveness, Adoption, Implementation, Maintenance/Sustainability extension framework for sustainability, implementation outcomes (reach, adoption, implementation, sustainability) will be evaluated using mixed methods.

**Discussion:**

This study represents one of the first studies to test the effectiveness of a culturally responsive family engagement intervention in CSC settings, where service utilization disparities among families are present. Findings from this study have the potential to improve the impact of CSC for families and advance understanding of equity-focused implementation approaches to facilitate the provision of culturally responsive care in mental health settings.

**Trial registration:**

ClinicalTrials.gov NCT06945055. Registered on March 24, 2025. https://clinicaltrials.gov/study/NCT06945055.

**Supplementary Information:**

The online version contains supplementary material available at 10.1186/s13063-025-09280-0.

## Background

Each year approximately 150,000 individuals will experience their first episode of psychosis (FEP). If left untreated, psychosis can have detrimental impacts leading to poorer quality of life and increased mortality [1–3]. FEP is often considered a critical window for intervention to alter the short- and long-term outcomes associated with schizophrenia-spectrum illnesses and other psychotic disorders [4, 5]. Coordinated specialty care (CSC) models (e.g., NAVIGATE, OnTrack, and EASA) are early intervention programs for FEP in the U.S. [6]. Key components of CSC include evidence-based and evidence-informed practices, such as individual psychotherapy, education and employment support, peer support services, medication management, and case management for those in the early stages of psychosis, and family education and support (e.g., family psychoeducation) [7–12]. The inclusion of family education and support as a core component of CSC is primarily due to the added benefit that family member involvement in care has on client engagement, symptom reduction, and relapse prevention [13–15]. Over the past decade, robust literature has demonstrated the effectiveness and positive impact of CSC for individuals in the early stages of psychosis and their family members throughout the U.S. [6, 9, 13, 16–21].

### Impact of family member engagement and gaps in early psychosis care

Family members play a key role in providing support and resources for young people with FEP and are often instrumental in the initiation of mental health services for their loved one [22–25]. Despite a large proportion of young people with FEP reside with a family member coupled with the benefits of having family members engage in services (e.g., improved client engagement and clinical outcomes), many CSC programs in the USA struggle to reach family members, especially those from historically underserved or minoritized communities (e.g., rural, ethnoracial minorities) [22, 26]. Prior research has found that approximately 50% of client’s family members participate in family education and support appointments embedded with CSC [21, 27, 28]. In addition, families from ethnoracially minoritized and low socioeconomic backgrounds are significantly less likely than non-Latinx white and high socioeconomic families to be involved in CSC [27–29]. Lack of family engagement has been associated with client disengagement, lower functional outcomes, and could potentially reduce program fidelity [30–32]. Considering that existing literature has amplified a critical need to improve the reach of CSC and engagement in services among family members, there have been no large-scale studies on family engagement interventions for CSC in the USA. Moreover, no studies have addressed disparities in engagement in CSC or other early intervention services for psychosis.

### Blending of disparities research and implementation science to facilitate culturally responsive care

Culturally responsive care requires mental health services or programs to (1) use patient-centered approaches; (2) practice culturally competent care that is responsive to clients’ cultural perspectives and backgrounds, which is often facilitated by the use of cultural assessment tools; and (3) practice cultural humility, which is the ability to identify one’s own biases and the willingness to learn from others as well as enter into a relationship with clients with the intention of honoring clients’ beliefs, customs, and values [33–36]. The use of cultural assessment tools helps mental health providers to understand salient cultural and contextual factors [37, 38], and has been associated with improved communication, leading to stronger rapport, and overall satisfaction with mental health services [37, 39–41]. In addition, structural competency training provides the knowledge and skills to understand stressors and social determinants of health (e.g., stressors associated with poverty, food insecurity, racism/discrimination, stress associated with immigration) that can have an impact on mental health and engagement [42]. Eliciting salient cultural and contextual information can guide the tailoring of appointments or sessions, improve quality of care, and reduce disparities [43]. Yet, prior research suggests only 19% of CSC programs integrate formal cultural or structural assessments, and there has been no literature documenting implementation in CSC models [44]. This study protocol addresses this gap and could lead to advancements in the use of equity-focused implementation strategies to support the integration of culturally responsive interventions in CSC, and more broadly, mental health settings.

### Research objectives and aims

Informed by the Health Equity Implementation Framework which explicitly focuses on equitable implementation of interventions to address inequities in care [45], the overarching goal of this study is to improve family engagement in CSC and address disparities in engagement. To accomplish this, the present study has three aims: (1) to evaluate whether integrating FAMES improves family member/support person engagement in CSC compared to an attention control condition (ACC); (2) to examine whether target mechanisms of connectedness, self-efficacy, and motivation mediate FAMES engagement among family members/support persons; and (3) to assess the impact of FAMES on reach, adoption, implementation, and maintenance using a concurrent mixed-methods approach.

## Methods

### Study design

**Fig. 1:**
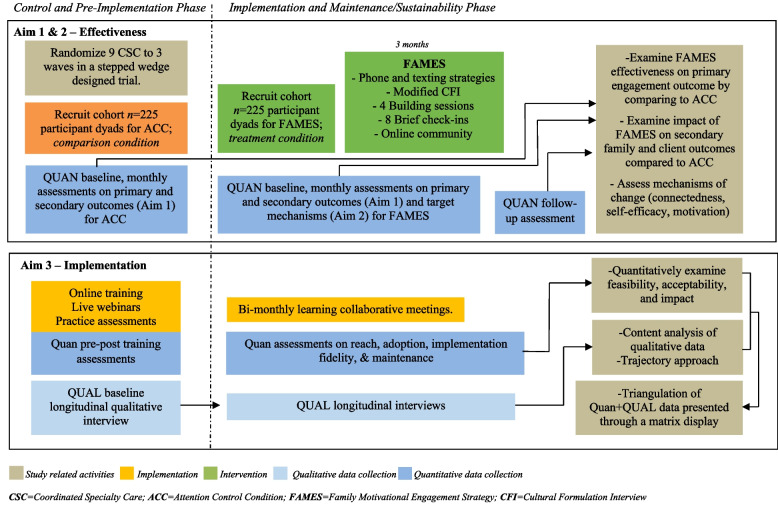
Overview of Hybrid Type 2 Effectiveness-Implementation Study Design

This Hybrid Type 2 Effectiveness-Implementation trial will use a cluster-randomized modified stepped-wedge design (see Fig. 1). Nine CSC sites throughout the USA, in both rural and urban regions, will be randomized to one of three start dates (waves) [46, 47]. Each site will recruit participant dyads, comprised of family member/support persons and clients with FEP enrolled in CSC, with separate cohorts of participant dyads recruited into the ACC and FAMES conditions. Aim 1 (effectiveness) seeks to evaluate whether integrating FAMES improves family engagement in CSC compared to ACC. In Aim 2 (mechanisms of change), we will use quantitative survey data to examine whether our theoretically driven target mechanisms of connectedness, self-efficacy, and motivation mediate FAMES engagement. Aim 3 (reach, adoption, implementation, and maintenance) will use a concurrent (quan + QUAL) mixed methods approach to evaluate implementation through an equity-focused lens. This study follows the Standard Protocol Items: Recommendations for Interventional Trials (SPIRIT) reporting guidelines (see Additional file 1 in the supplementary materials for the SPIRIT checklist) [48].

### Study sites

Nine CSC programs geographically distributed throughout the USA will serve as study sites. The geographical location of sites represents both rural and urban communities, hospital-based and community-based programs, and has a combined ethnoracially diverse set of families. CSC programs are generally comprised of a program director, family education and support specialist, peer support specialist, case manager, education and employment specialist, and a medical prescriber. Nine part-time family peers, with lived experience of having a loved one who has experienced psychosis, will be hired by each CSC at least three months prior to the pre-implementation phase. Across the nine CSC sites, the following team roles will be consented to participate in study activities: Program directors/supervisors (*n* = 4), family education and support providers (*n* = 4), program director/family education and support provider (*n* = 4), and family peers (*n* = 9).

### Randomization

**Fig. 2:**
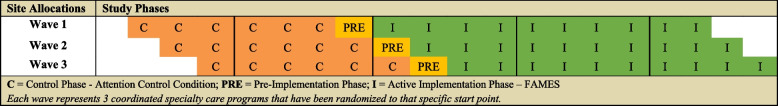
The modified stepped-wedge randomized trial across three waves. CSC, Coordinated Specialty Care; ACC, Attention Control Condition; FAMES, Family Motivational Engagement Strategy; CFI, Cultural Formulation Interview

CSC programs will be randomized to one of three waves with different study start times offset by three months (Fig. 2). A two-stage stratified randomization procedure will be applied to account for differences in site size (large vs. small), using a median split by site panel size to designate sites as large or small. This block randomization method stratified by the size of the CSC program will ensure small and large sites have a balanced opportunity to receive the innovation at any point in the study timeline. To reduce the possibility of implementation contamination, study staff will perform each randomization incrementally at six months prior to the start date for the next wave.

After site randomization, each CSC program will be allotted 18 months to recruit participant dyads to receive the ACC during the control phase, after which sites will participate in a three-month pre-implementation phase to commence training of family peers, program directors, and family education and support providers, followed by a 30-month active implementation phase to recruit a new cohort of participant dyads to receive FAMES (Fig. 2).

### Family member and client participant dyads

A total of 450 participant dyads will be recruited across nine CSC programs, with 225 participant dyads to receive the ACC and 225 to receive FAMES. Each participant dyad will include a family member and a loved one enrolled in CSC for FEP. Family member inclusion criteria are as follows: (1) one family member/support person of a client enrolled in CSC for ≤ 6 months; (2) ≥ 18 years of age; and (3) during the active implementation phase, did not participate in the ACC. Client inclusion criteria include the following: (1) ≥ 15 years of age, and (2) enrolled in CSC for ≤ 6 months. Potential participants will be excluded if they do not understand the consent and assent process, and if they are non-English or non-Spanish speaking adults or youth.

### FAMily Motivational Engagement Strategy (FAMES) intervention

FAMES uses context-based communication strategies to enhance the clinical encounter by targeting theoretically informed mechanisms to engage family members in CSC [49]. Participants in the FAMES condition will be assigned to a trained family peer who will deliver FAMES across 12 weeks. FAMES is intended to be a brief intervention consisting of weekly contact (i.e., phone, text messages, email) delivered across three phases: (1) Building, (2) Continuous Contact, and (3) Family Peer Connection Community.

During the Building Phase (weeks 1–4), the first two appointments, lasting approximately 30 min, place an emphasis on building trust and rapport by integrating a modified version of the Cultural Formulation Interview (CFI) to identify and discuss cultural and contextual factors unique to each family member [37]. Example questions include the following: “what aspects of their background or identity can make their experiences better or worse in this clinical setting and in the community?” or “what types of help, resources, treatment were useful and not useful?” or “are there any kind of stressors that make your experience worse, such as living environment, discrimination?” On a scale of 1 = not important to 10 = high importance family members will rate their perceived level of importance in four domains: (1) addressing challenges with their loved one, (2) finding ways to manage stressors, (3) integrating cultural identity into sessions, and (4) developing coping skills specific to culture. Family peers will then use the information gathered to assist family members with setting goals for future FAMES appointments and during CSC team meetings to inform family psychoeducation appointments with providers. The next two appointments focus on coping skills that match those identified using the CFI and communication skills that build competence and empower family members to advocate and share their needs with others.

The Continuous Contact Phase (weeks 5–12) consists of eight semi-structured brief check-ins, lasting approximately 20 min, to capitalize on the strength of family peer roles to engage families while providing the space for participants to raise topics of discussion most relevant to them, and inquire about domains of importance identified during the Building Phase [50]. As an extended component of CSC, information obtained by family peers during the Building and Continuous Contact Phases, will be shared within CSC team meetings and with family education and support providers to improve the relevancy of family psychoeducation sessions.

Family Peer Connection Community Phase will occur during weeks 10 and 12. Family member participants will be invited to attend virtual group sessions and be given access to a closed online FAMES Facebook group, facilitated by family peers from CSC sites, where they will be able to connect and share experiences with co-participants.

### CUlturally Responsive Approach to Targeting Equity (CURATE) implementation package

Informed by the Theoretical Domains Framework, CURATE involves a combination of implementation strategies and content to target four domains of behavior change [51–53]: knowledge, skills, social identity, and social influence. To target these domains, CURATE includes a combination of strategies and content integrated across the pre-implementation and active implementation phases [54, 55]. The implementation package will consist of four discrete implementation strategies (distribution of educational materials, ongoing training, creating a learning collaborative, and auditing and providing feedback) from the Expert Recommendations for Implementing Change (ERIC) taxonomy and two persuasive communication approaches (storytelling and photovoice), often used in disparities research [56–59].

#### Pre-implementation phase

Educational materials on FAMES (e.g., guide, checklists) will be distributed electronically and by mail to family peers, program directors, and family education and support providers. An online digital learning platform will house a document library consisting of FAMES educational materials, digital storytelling videos sharing experiences from diverse family members, narrated videos outlining the delivery of FAMES, as well as how-to guides. The online learning environment will facilitate ongoing training across five weeks consisting of assigned weekly written and pre-recorded modules and weekly 90-min virtual training for discussion of assigned modules. Modules 1 and 2 that will embed equity-driven content (i.e., positionality, structural competency) and incorporate storytelling and community-based photovoice activities focused on understanding personal qualities that influence clinical encounters with family members [60, 61]. Modules 3 and 4, which cover components and delivery of FAMES include the use of the modified CFI, conceptualization, and integration within CSC. Module 5 will outline fidelity and monitoring of FAMES. Program directors and family education and support specialists will complete the self-paced online weekly modules. Family peers will complete the online modules and participate in the weekly virtual training sessions.

Following the 5-week didactic training, each family peer will complete two practice assessments over the course of two weeks with “participant actors” who will be pre-identified individuals with family members’ lived experience from diverse backgrounds (e.g., ethnoracial group, spirituality). Family peers will receive immediate feedback after each practice assessment from participant actors and two members of the study team, as well as written feedback in the form of a fidelity summary outlining skillfulness, adherence, and integration with CSC.

#### Active-implementation phase

A virtual learning collaborative will be created across all sites consisting of family peers. Learning collaborative meetings will be held bi-monthly with two members of the study team to provide a space for support and encouragement from family peers and to share successes and implementation barriers. Each meeting will also include specific learning topics (e.g., burnout, secondary trauma, cultural considerations) to facilitate ongoing training.

### Attention control condition

Family member participants in the ACC will receive weekly automated messages for 12 weeks based on participant communication preference (email, text message). Messages will include positively framed messaging informed by other text messaging-based studies [62, 63], along with educational materials about psychosis, tips (e.g., communication, de-escalation), a list of community-based and online resources and events, and reminder messages for upcoming appointments. Automated messages are outlined in Additional file 2 in the Supplementary Materials.

### Participant recruitment

CSC staff at each site will place recruitment materials in waiting rooms, within intake packets presented to clients and family members, and presented to all families enrolled in services no longer than 6 months. Interested family member participants may contact the research team directly or inform CSC staff of their interest. Research staff will also contact family members who have given CSC staff permission to share contact information. All potential participants will be screened by research staff for eligibility and those who are not eligible will be provided with a list of peer resources available in their community and online. Research staff will review and electronically send the consent form via the Research Electronic Data Capture (REDCap) e-consenting platform to all eligible participants by phone or video conference.

### Data collection procedures

**Fig. 3:**
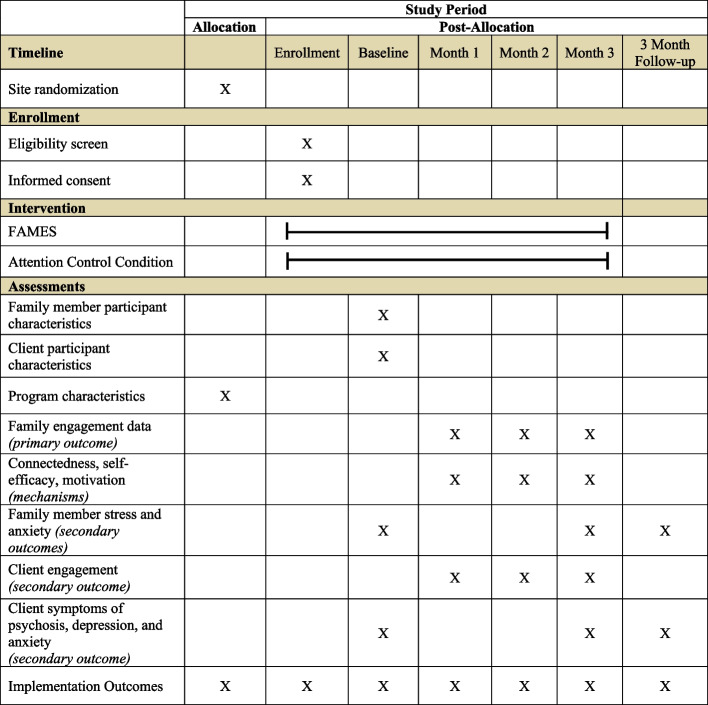
SPIRIT diagram: schedule of enrollment, interventions, and assessments

Family members in the ACC and FAMES conditions will complete quantitative assessments at baseline, months 1–3, and at follow-up (3 months post-intervention), whereas client participants will complete assessments at baseline, month 3, and at follow-up. Figure 3 displays the SPIRIT diagram outlining the schedule of measures across the study period. All assessments will be collected by research staff over the phone and entered directly into REDCap. For participants who prefer to complete assessments on their own, SMS or link-to-text survey features in REDCap will be enabled to capture responses. For completing all assessments, family member/support person participants will receive a total of $275 and client participants will receive a total of $110.

Table 1 provides a description of all study measures completed by participant dyads to assess primary and secondary outcomes and mechanisms of change. Measures with accessible versions in the Spanish language will be used, and if not, will be translated into Spanish.

**Table 1:** Effectiveness outcomes: study assessments for primary, secondary, and mechanisms

Construct	Measure	Data Source	Timepoint
*Primary outcome*	**Engagement in CSC Family Psychoeducation (Aim 1)** will be measured as first session attendance rate and total attendance rate per family	Administrative data	Monthly
	**Engagement in FAMES (Aim 2)** will be measured by FAMES session attendance rate and the total number of contact minutes with participants by videoconference, in-person, and phone, and # of encounters by email, and text	Family peer survey	Weekly
*Secondary family outcomes*	**Perceived Stress (Aim 1)** will be assessed using the *Perceived Stress Scale*, which contains 10 Likert-style items to assess the degree to which an individual appraises life as stressful	Family participant survey	BL, 3 m, FU
	**Anxiety (Aim 1)** will be assessed using the *General Anxiety Disorder-7 item* [64]*.* Scores 8 or greater is cut-off for anxiety disorder	Family and client participant survey	BL, 3 m, FU
*Secondary client outcomes*	**Client Engagement in CSC (Aim 1)** will be measured as the client attendance rate in CSC	Administrative data	Monthly
	**Depression (Aim 1)** will be measured using the *Patient Health Questionnaire-9 item* [65]. A score of 10 or greater is cut-off for depression	Client participant survey	BL, 3 m, FU
	**Psychosis (Aim 1)** will be measured using *Community Assessment of Psychic Experiences – Positive 15-items Scale* [66]	Client participant survey	BL, 3 m, FU
*Mechanisms*	**Self-Efficacy (Aim 2)** will be measured using the 10-item *General Self-Efficacy scale* [67]. Scores range from 10 to 40, with higher scores indicating greater self-efficacy	Family participant survey	BL, Monthly
	**Connectedness (Aim 2)** will be assessed using the *Social Connectedness subscale of YSS-F* [68, 69], which assesses social support. Scores range from 4–20 where higher scores indicate greater social connectedness	Family participant survey	BL, Monthly
	**Motivation (Aim 2)** will be assess using the 26-item *Treatment Motivation Questionnaire t*o assess motivation about services	Family participant survey	BL, Monthly
**Other indicators (to be used as covariates/independent variables in analyses if Aims 1–2)**			
*Individual characteristics*	**Family member and client characteristics** will be assessed using modules from the *PhenX Toolkit Social Determinants of Health Core and social determinants of health*. Age, gender identity, sex, race, ethnicity, education, employment, marital status, religious preference, health insurance, etc	Family & client participant survey	BL
*Contextual factors*	**Contextual factors** will be assessed using the *Citizenship Measure* is a 46-item that measures community connectedness and engagement [70]	Family participant survey	BL
*Program characteristics*	**Program characteristics** will include setting, location, # of team members	Provider survey	BL

*BL *baseline, *PRE *pre-implementation phase, *FU *3-month post follow-up

Implementation outcomes are guided by the extension Reach, Effectiveness, Adoption, Implementation, Maintenance/Sustainability (RE-AIM) extension framework which identifies indicators related to sustainability and health equity-related considerations for tracking each domain of RE-AIM [71–74]. Table 2 outlines all measures to capture implementation outcomes which will be completed by family peers and CSC providers (i.e., family peers, program directors, and family education and support providers). Research staff will email participating CSC providers a unique REDCap link to complete pre- and post-training surveys during the pre-implementation phase. A longitudinal qualitative research approach will be used to explore potential barriers and facilitators to delivery and engagement that may inform modifications to implementation over time and understand experiences over time during the active implementation phase [77–79]. Semi-structured qualitative interviews will be scheduled and conducted via videoconference (i.e., Zoom) with family peers at baseline (end of pre-implementation phase) and at 6, 12, and 24 months after the commencement of the active implementation phase. Interviews will also be conducted with family education and support providers and/or program directors at baseline and at 12 and 24 months after the commencement of the active implementation phase. All interviews will be audio recorded, transcribed verbatim, and reviewed for accuracy.

**Table 2:** Implementation outcomes: study assessments

Construct	Measure	Data source	Timepoint
*Implementation outcomes*	**Reach of FAMES (Aim 3)** will be measured as the % (and number) of participants who enroll in FAMES out of the # of service users identified at intake. Reach will be categorized by demographic characteristics (e.g., race, ethnicity, SES, distance from CSC). Interviews with family peers and providers will assess potential reasons for stronger or weaker reach	Provider survey, administrative data, and interviews	Monthly
	**Adoption (Aim 3)** will be measured as the % of family psychoeducation sessions that integrate information gathered from FAMES	Provider survey	Monthly
	**Competence of Family Peers (Aim 3)** will be assessed using five-item rater scale developed by the team and informed by previous research [75] for such topics as active listening, exploring barriers to engagement, incorporating prior information into current session, setting goals	Practice assessment and FAMES sessions	PRE, 12 m, 18 m, 24 m
	**Acceptability (Aim 3)** will be assessed using a feedback survey with multiple and short answer response items on satisfaction, will be administered after each live webinar session	Family peer and provider surveys	PRE
	**Knowledge (Aim 3)** will be assessed using a developed 10-item pre- and post- assessment embedded in the online training platform	Family peer and provider surveys	PRE
	**Implementation adaptations, barriers, and facilitators (Aim 3)** will be identified from longitudinal qualitative interviews	Family peer and provider interviews	PRE, 6 m, 12 m, 24 m
	**Sustainability of FAMES (Aim 3)** will be measured with the *Program Sustainability Assessment Tool* a 40-item scale to assess ongoing program support, funding capacity, and fit [76]	Provider survey, family peer and provider interviews	FU
**Other indicators (to be used as covariates/independent variables in analyses if Aims 3)**			
*Individual characteristics*	**Family peer and provider characteristics** will include role, education level, month/years of experience, race, ethnicity, sex, gender identity	Family peer, and provider survey	BL
*Program characteristics*	**Program characteristics** will include setting, location, # of team members	Provider survey	BL

*BL *baseline, *PRE *pre-implementation phase, *FU *3-month post follow-up

### Data analysis plan

All analyses will follow an intention-to-treat principle with participant data within clusters analyzed according to the cluster’s randomized crossover time. All quantitative analyses will utilize a type I error rate of *p* < 0.05. Multiple imputation will be used for missing secondary psychological outcomes, with separate imputations conducted by study phase and clinic, incorporating demographic variables to ensure the accuracy of imputed values under the assumption of missing at random. Analyses will be conducted using STATA version 14.2, R statistical software, or the Statistical Package for the Social Sciences (SPSS, version 31.0).

#### Aim 1: Impact of FAMES on primary and secondary outcomes

##### Quantitative data analysis

Statistical analyses for primary engagement outcomes will use Generalized Linear Mixed Models (GLMM) to compare engagement rates between the control (ACC) and intervention (FAMES), with a random effect for cluster (site), a fixed effect for each phase (ACC and FAMES), and a fixed effect for implementation wave to control potential time-related confounding [80, 81]. GLMM will also be used to assess change scores on secondary outcomes for family member and client participants from baseline to 3 months. Restricted maximum likelihood estimation with Kenward-Roger standard error and degrees of freedom correction to accommodate for our relatively small number of clusters will be used. Supplemental analyses will assess the likelihood of including relevant individual and program covariates (e.g., age, biological sex, race, and others) in models if significant differences among covariates occur between ACC and FAMES samples. Analysis of the secondary outcomes collected only from FAMES participants at follow-up will employ GLMMs to compare baseline to 3-month to follow-up inventory scores as a random effect repeated measures factor. Fixed effect covariates (e.g., age, biological sex, race, and others) will be included in these models as appropriate. A Bonferroni correction will be applied to primary engagement outcomes (first session attendance and total attendance per family and client) to control for multiple testing.

#### Aim 2: Assess whether target mechanisms mediate FAMES engagement

##### Quantitative data analysis

To examine whether potential mechanisms (connectedness, self-efficacy, and motivation) mediate engagement in FAMES, hierarchical linear regression (HLR) will be used, with predictor variables entered in sequential steps. Demographic variables (age, gender, biological sex, race, ethnicity, years of education, employment status, marital status, clinic), will be entered in Block 1, followed by changes in mediator variables in Block 2. The unique contribution of each mediator will be assessed using changes in adjusted R2, and only significant predictors will be retained in the final models. Regression assumptions will be evaluated using variance inflation factors (multicollinearity) and Durbin-Watson statistics (independence of errors). To explore potential nonlinearity, alternative specifications such as interaction terms or polynomial transformations may be tested.

#### Aim 3: Explore implementation outcomes using mixed methods

##### Quantitative data analysis

All implementation outcomes will be analyzed using GLMM to account for clinic- and provider-level clustering and repeated measurements over time. The models will include random effects for sites and providers and fixed effects for time (such as monthly changes in reach and adoption). Since provider characteristics such as gender, race, and education may influence implementation, they will be included in models if they improve accuracy, as assessed by Akaike Information Criterion (AIC) and Bayesian Information Criterion (BIC). Because some providers may drop out or miss assessments, multiple imputation (MI) will be used to estimate missing data, ensuring unbiased results.

##### Longitudinal qualitative data analysis

A conventional content analysis approach will be used to initially code transcripts from CSC team members. Three members of the research team will independently read and analyze 20% of transcripts, developing codes and themes that come from the data prior to reaching consensus. To guard against biases, an audit trail documenting analytical decisions will be created and peer debriefing meetings will be held. Meeting discussions and notes will be used to develop a final code book. The remaining transcripts will be coded by trained research staff. ATLAS.ti software will be used to assist with analysis [82]. A trajectory analysis approach will be used to understand experiences over time, when the same cohort can be maintained that can then be compared [83, 84]. Codes will be organized into time-ordered sequential matrices per site. Codes deriving from transcripts will be entered into a series of matrices organized by themes and time points by sites and across sites.

##### Mixed methods integration

The comparison and integration of findings from quantitative and qualitative data will be used to better understand metrics for reach, implementation fidelity, and maintenance. Merging of data will occur through triangulation which is often used to display findings emerging from different data types to gain a better understanding or a more complete picture [85]. We will develop a matrix to assess whether findings from quantitative and qualitative data converge (provide the same answer), contradict each other (dissonance), or are salient [86]. Results from our qualitative analysis will also be embedded within the quantitative data to provide context for outcomes (e.g., how, why?) using a complementary approach.

### Power analysis

All power analyses were conducted with G*Power v. 3.1 [87], using different potential sample and effect sizes, assuming nine CSC sites, and three waves to estimate the power to detect effects. Power estimations for Aim 1 focused on engagement primary outcomes. Using the design effect approach in the Shiny-CRT power estimation application [88], a sample size of 450 participants, with an effect size of 0.5, at a 2-sided alpha level of 0.025, and assuming an ICC in each period of 0.01, the estimated power to detect a significant difference between ACC and FAMES conditions for the primary outcome of engagement would exceed 85%. For specific to Aim 2, power analyses assumed a conservative *R*^2^ value of 0.10 for predicting engagement from seven Block 1 control variables. Based on a sample of 225 family member participants in the FAMES condition, at a two-sided alpha level of 0.05, estimated power would be 98% when assessing whether *R*^2^ differed significantly from zero. With the addition of three Block 2 mediator variables (change in inventory scores), if *R*^2^ was to increase to 0.20 so that the change in *R*^2^ is 0.10, with an adjustment to the two-sided alpha to 0.025 due to multiple hypothesis tests and potential correlation between Block 1 and 2 predictors, and an adjusted effect size for Block 2 change given Block 1 variables [89] power would increase to 99% to detect a significant change in *R*^2^. Estimated power to achieve significant differences in implementation outcomes (i.e., pre- and post-change in knowledge) assumed a standardized mean difference of 0.60. Based on a sample of 20 family peer and provider participants, at a one-sided alpha level of 0.05, and a correlation among repeated measures of 0.50, estimated power would be 83% to establish significant change in knowledge. As such, this study is sufficiently powered (> 80% power) to detect meaningful differences in engagement outcomes, mediation effects, and knowledge, with adjustments for clustering and the stepped-wedge design structure.

### Data monitoring and management plan

All quantitative data will be collected via secure Health Insurance Portability and Accountability Act (HIPAA)-compliant REDCap housed by Washington State University, Spokane. Qualitative transcripts will be de-identified and all qualitative data will be stored on a secure HIPAA-compliant server. All procedures have been approved by the Institutional Review Board (IRB) at Washington State University. All amendments to the protocol will be submitted to Washington State University’s IRB. An external data safety monitoring board (DSMB) has been convened and consists of three experts in statistical analyses, disparities research, and early psychosis, respectively, who will monitor adverse events to ensure the safety and integrity of the data, in compliance with requirements set by the National Institute of Health (NIH). Due to the minimal risk associated with this study, the DSMB will meet annually to review a summary report of adverse events and recruitment progress yearly. Summary of reports, along with DSMB recommendations will be submitted to NIH as part of the yearly progress reports. Consistent with reporting standards by the National Institute of Mental Health, de-identified data will be submitted to the National Data Achieves (NDA) two times per year, and a final de-identified dataset will be available and accessible from the NDA, upon request. This study was registered with ClinicalTrials.gov (#NCT06945055).

### Dissemination plan

Study findings will be disseminated through peer-reviewed manuscripts submitted to academic journals, presentations at academic conferences, and community presentations (e.g., presentations to CSC agencies, state mental health authorities). Standardized guidelines for authorship, such as the International Committee of Medical Journal Editors, will be followed. According to NIH policies, all peer-reviewed manuscripts will be submitted to PubMed Central for open access. In addition, results from the present study will be submitted to ClinicalTrials.gov no later than 12 months after the conclusion of the study.

## Discussion

Family member or support person involvement across the continuum of care for early psychosis is vital. While there is a relatively large and growing body of literature on the impact of CSC for those experiencing their first episode of psychosis [6, 9, 13, 16–21], many programs struggle to engage family members [21, 22, 27, 28]. Ethnoracial and geographic disparities in family engagement also exist and have been associated with individual-level (e.g., conflicts, transportation) and program-level barriers (e.g., cultural fit, hours of operation) [90]. As such, this study addresses a significant gap in strategies to improve family engagement in CSC programs, while simultaneously mitigating known disparities. By pairing FAMES, a culturally responsive engagement intervention, with CURATE, an implementation package specifically designed to focus on equity and providing culturally responsive care, this Hybrid Type 2 study will advance our understanding of equity in the context of developing an implementation package and effective family engagement interventions that if successful, can be deployed and scaled up in CSC settings.

## Trial status

Protocol version #3, approval received on the 6th of August 2025. Participant recruitment for this study began in May 2025 and is expected to conclude in October 2029.

## Supplementary Information

Supplementary Material 1

Supplementary Material 2

## Data Availability

Deidentified data are submitted to the National Institute of Mental Health (NIMH) Data Archive twice per year and will be made publicly available two years following the completion of the study, per their requirements.

## Abbreviations

ACC: Attention control condition
CSC: Coordinated specialty care
CFI: Cultural Formulation Interview
CURATE: Culturally Responsive Approach to Targeting Equity
DSMB: Data safety monitoring board
ERIC: Expert Recommendations for Implementing Change taxonomy
FAMES: Family Motivational Engagement Strategy
FEP: First episode psychosis
IRB: Institutional Review Board
NDA: National Data Achieves
NIH: National Institute of Health
RAISE-ETP: Recovery After an Initial Schizophrenia Episode-Early Treatment Program
RE-AIM: Reach, Effectiveness, Adoption, Implementation, and Maintenance/sustainability
REDCap: Research Electronic Data Capture
