# Supplementary material for: FAMily Motivational Engagement Strategy (FAMES) for coordinated specialty care programs: study protocol to evaluate a culturally responsive engagement intervention and equity focused implementation strategies in a hybrid type 2 randomized stepped-wedge trial
Source: Trials. 2025 Dec 16;26:568. doi: 10.1186/s13063-025-09280-0 (PMC12709835; doi:10.1186/s13063-025-09280-0)
Supplement: Supplementary file 2 — Supplementary Material 2 [file 13063_2025_9280_MOESM2_ESM.docx]

**Supplementary Materials 2**: Attention Control Condition Automated Messages

| **Week** | **Automated Messages** |
| --- | --- |
| **1** | Hello [name], I hope you are well! Thank you for joining the FAMES study. We understand that this period of time can be stressful and confusing. Remember, psychosis is nobody's fault - people do not cause it, but it is treatable and many individuals who experience a psychotic episode go on to live successful lives. This is improved by having the support of their loved ones. Thank you for being a support to your loved one as they go through this difficult time. If you have any questions or concerns about how being involved in your loved one's care may influence their outcomes, please reach out to your family psychoeducation specialist. - FAMES |
| **2** | Hi [Name], Do you have a notebook that you take to your family sessions? Sometimes questions come up that we forget about before we have a chance to meet with our clinicians, or we want to take notes while we are meeting with our clinicians. A notebook just for these sessions can be a useful way of making sure that you get what you need out of your clinicians. It is encouraged to come to your session with questions and to write down notes. - FAMES |
| **3** | Hi [Name], sometimes you just need to talk to someone about your mental health. Warmlines are free and confidential numbers you can call staffed by peers who can provide support when you just need someone to talk to. To find a warmline today by visiting https://www.warmline.org/#directory - FAMES |
| **4** | Hi [name], managing our own stresses can often be forgotten when taking care of the needs of others. Take this moment to practice a breathing coping skill called 'Take 5 - Triangle Breathing'. First, pause and reflect on your well-being and breathe. Breathe in for 5 seconds. Hold that breath for 5 seconds. Breathe out for 5 seconds. Try triangle breathing for 1 minute at least once a day. - FAMES |
| **5** | Hey [name], just a reminder that family conflict during this time is normal, but don't forget to take the time to take a step back and find at least one thing you enjoy today. That could be taking a short walk around the block, listening to your favourite song, reading a chapter of a book, gardening, whatever makes you feel at peace. As we recenter ourselves we can come back to our loved ones in a calmer, more productive way. You are worth making time for. - FAMES |
| **6** | We know that life can difficult sometimes, just know that you have support. Feel free to connect with your family education and support person if you are looking for additional resources and support for you. Sending you positive vibes this week. - FAMES |
| **7** | Hi [Name], NAMI (the National Alliance on Mental Illness), is an organization located across the U.S. that offers free support and education. NAMI Family Support Group is a peer-led group for any adult with a loved one who is experiencing a mental illness that is free, confidential, and meets weekly or monthly. Check out their website today: https://www.nami.org/ - FAMES |
| **8** | Hello [name], it's okay to not be okay. While it feels like you should be focusing your energy on your loved one, keeping things positive, and managing all of life's stress, it is normal to need and want some additional support. Psychology Today is a website where you can find therapists (online and in person), psychiatrists, treatment centers, and support groups. It allows you to filter by geographic region, gender, specialty, ethnicity, sexuality, insurance, language, and more. Open the link today and see what is available to support you https://www.psychologytoday.com/us. - FAMES |
| **9** | Hello [Name], Don't forget yourself when thinking of things you have to do today. Pay attention to your own needs and feelings. Engage in a task that you find enjoyable, even if only for a few minutes. Your needs are important too. - FAMES |
| **10** | Hey [Name], during this period of time, you may be looking for additional resources and to connect with others. Have you heard of the Schizophrenia and Psychosis Action Alliance (https://sczaction.org/) or Mental Health America (https://mhanational.org/)? Both of these organizations have materials and online communities so you can learn more about mental health treatment, psychotic disorders, and resources near you. - FAMES |
| **11** | Hi [Name], It can be hard to think about the way things were before your loved one experienced their mental health concern. It is okay to be stressed and frustrated, but remember, people experiencing psychosis do recover. Recovery is not a straight line, and there may be times where things feel hopeless and like they are moving backwards. Talk to your clinicians and peer groups who can help support you during these times and remind you that things will get better. - FAMES |
| **12** | Hello [Name], Thank you for participating in FAMES. Remember, it is encouraged to ask your clinicians questions and to take notes. Find additional resources like NAMI, Mental Health America, Psychology Today, and the Schizophrenia and Psychosis Action Alliance who can provide additional knowledge and support groups. If you and your loved are ever in a crisis, dial or 988, to get connected to your local crisis line. They are available 24/7 every day of the year. Lastly, take time for yourself. Mental health challenges do not happen in a vacuum, and it is a positive thing to take time for yourself and find support for you as you navigate these moments. - FAMES |
